# For Intestinal Antisepsis in Typhoid

**Published:** 1900-08

**Authors:** 


### For Intestinal Antisepsis in Typhoid.

Rozster (*Gazzetta degli ospedali e delle cliniche; Gazzetta medica lombarda,* April 15th) recommends the following:

℞ Thymol,
Carbonate of guaiacol, } of each....90 grains.
Medicinal soap,

M. Divide into thirty powders, of which one may be taken every four hours.—*N. Y. Medical Journal.*

